# Crystallization and preliminary X-ray diffraction analyses of the redox-controlled complex of terminal oxygenase and ferredoxin components in the Rieske nonhaem iron oxygenase carbazole 1,9a-dioxygenase

**DOI:** 10.1107/S2053230X14018779

**Published:** 2014-09-25

**Authors:** Jun Matsuzawa, Hiroki Aikawa, Takashi Umeda, Yuji Ashikawa, Chiho Suzuki-Minakuchi, Yoshiaki Kawano, Zui Fujimoto, Kazunori Okada, Hisakazu Yamane, Hideaki Nojiri

**Affiliations:** a Biotechnology Research Center, The University of Tokyo, 1-1-1 Yayoi, Bunkyo-ku, Tokyo 113-8657, Japan; b Education and Research Support Section,Technology Management Division, Administration and Technology Management Center for Science and Engineering, Waseda University, 3-4-1 Okubo, Shinjuku-ku, Tokyo 169-8555, Japan; c SR Life Science Instrumentation Unit, Research Infrastructure Group, Advanced Photon Technology Division, RIKEN SPring-8 Center, RIKEN Harima Branch, 1-1-1 Kouto, Sayo-cho, Sayo-gun, Hyogo 679-5148, Japan; d Biomolecular Research Unit, National Institute of Agrobiological Sciences, 2-1-2 Kannondai, Tsukuba, Ibaraki 305-8602, Japan; e Department of Biosciences, Teikyo University, 1-1 Toyosatodai, Utsunomiya, Tochigi 320-0003, Japan

**Keywords:** Rieske nonhaem iron oxygenase, electron-transfer complex, terminal oxygenase, ferredoxin, carbazole 1,9a-dioxygenase

## Abstract

The initial reaction in bacterial carbazole degradation is catalyzed by carbazole 1,9a-dioxygenase, which consists of terminal oxygenase (Oxy), ferredoxin (Fd) and ferredoxin reductase components. The electron-transfer complex between reduced Oxy and oxidized Fd was crystallized at 293 K using the hanging-drop vapour-diffusion method with PEG 3350 as the precipitant under anaerobic conditions. The crystal diffracted to a maximum resolution of 2.25 Å and belonged to space group *P*2_1_, with unit-cell parameters *a* = 97.3, *b* = 81.6, *c* = 116.2 Å, α = γ = 90, β = 100.1°. The *V*_M_ value is 2.85 Å^3^ Da^−1^, indicating a solvent content of 56.8%.

## Abstract 2

A crystal was obtained of the complex between reduced terminal oxygenase and oxidized ferredoxin components of carbazole 1,9a-dioxygenase. The crystal belonged to space group *P*2_1_ and diffracted to 2.25 Å resolution.

## 1. Introduction   

Rieske nonhaem iron oxygenases (ROs) play an important role as the initial enzymes in aromatic compound catabolic pathways (Gibson & Parales, 2000 ▶; Parales & Resnick, 2006 ▶). ROs consist of two or three discrete soluble components. Two-component ROs consist of reductase (Red) and terminal oxygenase (Oxy) components, whereas three-component ROs consist of Red, ferredoxin (Fd) and Oxy components. Oxy invariably contains a Rieske-type [2Fe–2S] cluster and a nonhaem iron active centre involved in dioxygen activation and it catalyzes the incorporation of molecular dioxygen into the substrate. Although there are variations in the redox-transfer machineries in both Fd and Red, these electron-transfer components transfer electrons from NAD(P)H to Oxy.

Carbazole 1,9a-dioxygenase (CARDO) was originally isolated from *Pseudomonas resinovorans* CA10 as the initial enzyme of the carbazole-degradation pathway, and similar degradation systems have subsequently been reported in several other bacteria, for example *Nocardioides aromaticivorans* IC177, *Novosphingobium* sp. KA1 and *Janthinobacterium* sp. J3 (Nojiri & Omori, 2006 ▶; Nojiri, 2012 ▶; Inoue & Nojiri, 2014 ▶). The amino-acid sequences of CARDO components from CA10 are nearly identical to those of J3, and the components can replace each other. Therefore, using the CARDO components from CA10 and J3, we have evaluated the reaction cycle and the electron-transfer mechanism among components in RO, especially between Fd and Oxy, by determining the crystal structures of the oxidized forms of Fd from CA10 (Nam *et al.*, 2005 ▶) and Oxy from J3 (Nojiri *et al.*, 2005 ▶) and of the complex forms of Oxy from J3 and Fd from CA10 (Ashikawa *et al.*, 2006 ▶). Recently, substrate-bound and oxygen-bound forms of the Oxy–Fd complex were determined and the catalytic cycle of Oxy was elucidated from a structural viewpoint (Ashikawa *et al.*, 2012 ▶). Crystals of Oxy alone, Fd alone and Oxy–Fd complexes and the conditions for their formation are summarized in Table 1 ▶.

**Figure 1:**
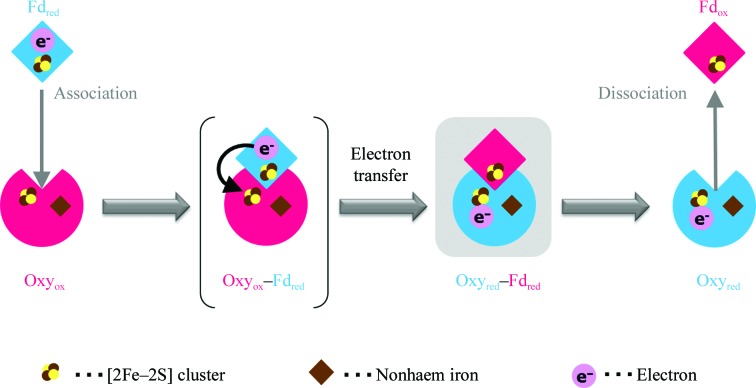
The electron-transfer reaction between the Oxy and Fd components of CARDO. Reduced and oxidized states of the components are shown in blue and red, respectively. Electrons are shown as pink spheres labelled e^−^. The black arrow in the proposed complex state (Oxy_ox_–Fd_red_) in parentheses shows electron transfer from the Rieske-type [2Fe–2S] cluster of Fd_red_ to the Rieske-type [2Fe–2S] cluster of Oxy_ox_. The Oxy_red_–Fd_ox_ complex obtained in this study is shown against a grey background.

Electron transfer between Oxy and Fd components is proposed to proceed in three steps (Fig. 1 ▶). Firstly, Fd_red_ associates with Oxy_ox_ (where the subscripts ‘ox’ and ‘red’ indicate the oxidized and reduced states, respectively). Next, the electron is transferred from Fd_red_ to Oxy_ox_ and the redox states of the components are reverted. Finally, Fd_ox_ dissociates from Oxy_red_. To accomplish these sequential steps effectively, the manner of interaction between Oxy and Fd should be altered according to the redox states of the respective components. This is supported by the observation that redox-state-dependent structural change triggers the association/dissociation of the Red and Fd components of biphenyl 2,3-dioxygenase (Senda *et al.*, 2007 ▶). However, our previously solved complex structures of Oxy and Fd, of both oxidized Oxy_ox_–Fd_ox_ and reduced Oxy_red_–Fd_red_ (Ashikawa *et al.*, 2006 ▶), do not appear in the catalytic cycle of the CARDO reaction; however, the structures clearly revealed the binding region between the two components. Clarification of the manner of interaction between Oxy_red_ and Fd_ox_ or between Oxy_ox_ and Fd_red_ would facilitate an understanding of the redox-dependent association/dissociation mechanism between Oxy and Fd components in RO based on comparisons among the structures in the actual catalytic cycle. Here, we report crystallization and preliminary X-ray diffraction studies of the complex crystal structures of Oxy_red_ and Fd_ox_ in CARDO.

## 2. Methods and results   

### 2.1. Purification, anaerobic procedure and protein reduction   

The Oxy component of CARDO from *Janthinobacterium* sp. J3 and the Fd component of CARDO from *P. resinovorans* CA10 were purified as described previously (Ashikawa *et al.*, 2005 ▶; Matsuzawa *et al.*, 2013 ▶). Briefly, histidine-tagged Oxy and Fd were expressed in *Escherichia coli* BL21(DE3) (Novagen, Madison, Wisconsin, USA) and purified using metal-chelation chromatography followed by gel-filtration chromatography. After purification, Oxy and Fd were subjected to ultrafiltration and buffer-exchanged into 50 m*M* Tris–HCl pH 7.5 using Vivaspin 20 membranes (10 000 MWCO; Sartorius, Göttingen, Germany) and Centriprep YM-10 (Millipore, Bedford, Massachusetts, USA), respectively. These protein solutions were flash-frozen in liquid nitrogen and stored at 193 K.

To prepare the Oxy and Fd solutions for the anaerobic crystallization experiments, an anaerobic chamber filled with 95% N_2_ and 5% H_2_ was used as described previously (Matsuzawa *et al.*, 2013 ▶). Purified Oxy and Fd were put into the anaerobic chamber and incubated for several hours on ice to remove the dissolved oxygen. Oxy was then reduced using two equivalents of sodium dithionite dissolved in deoxygenated 50 m*M* Tris–HCl pH 7.5. Fd and reduced Oxy were separately concentrated and buffer-exchanged four times into deoxygenated 50 m*M* Tris using Nanosep 10K Omega devices (Pall, Port Washington, New York, USA) to eliminate the dissolved oxygen and remaining sodium dithionite (in the case of Oxy). Aliquots of the protein solutions were subsequently added to individual quartz cells sealed with butyl rubber caps and the redox states were confirmed by measuring the absorption spectra as described previously (Matsuzawa *et al.*, 2013 ▶). Oxy and Fd showed the peaks characteristic of the redox states of the Rieske [2Fe–2S] cluster: Oxy_red_, 420 and 525 nm; Oxy_ox_, 459 and 560 nm; Fd_red_, 432 and 515 nm; and Fd_ox_, 457 and 570 nm (Nam *et al.*, 2002 ▶). Protein concentrations were estimated using a protein assay kit (Bio-Rad, Richmond, California, USA) with BSA as the standard.

### 2.2. Crystallization   

**Figure 2:**
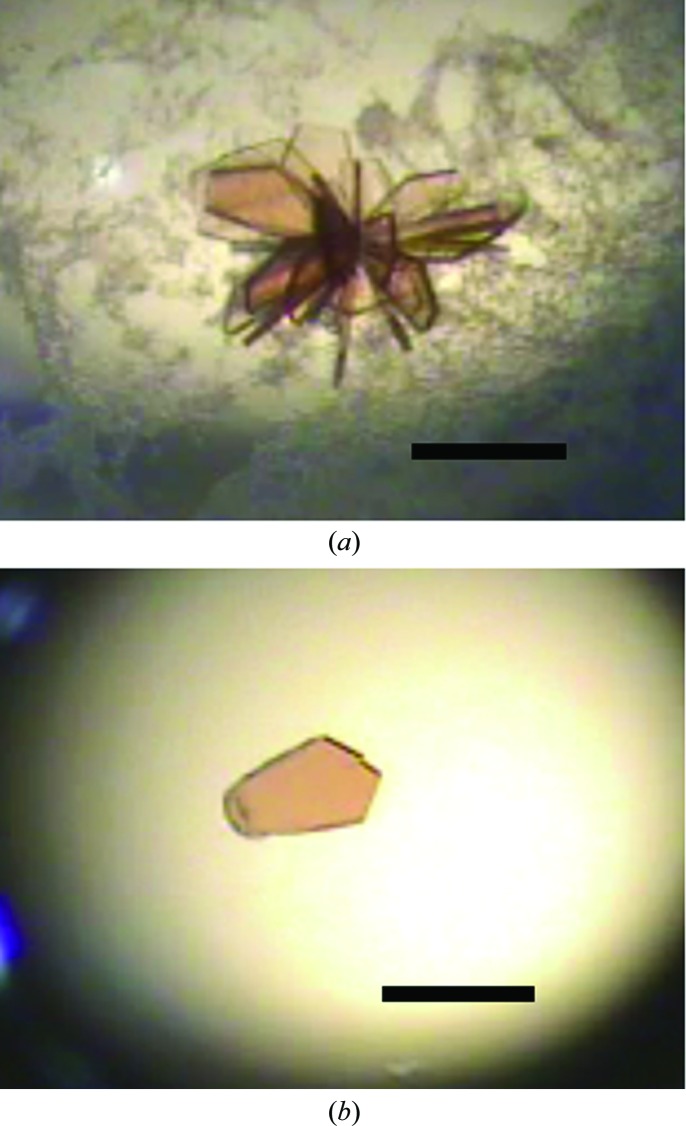
Crystals of the binary complex between the reduced state of Oxy from *Janthinobacterium* sp. J3 and the oxidized state of Fd from *P. resinovorans* CA10. Plate-shaped crystals in the drop (*a*) and one piece of the crystal (*b*) are shown. The scale bar is 0.3 mm in length.

**Figure 3:**
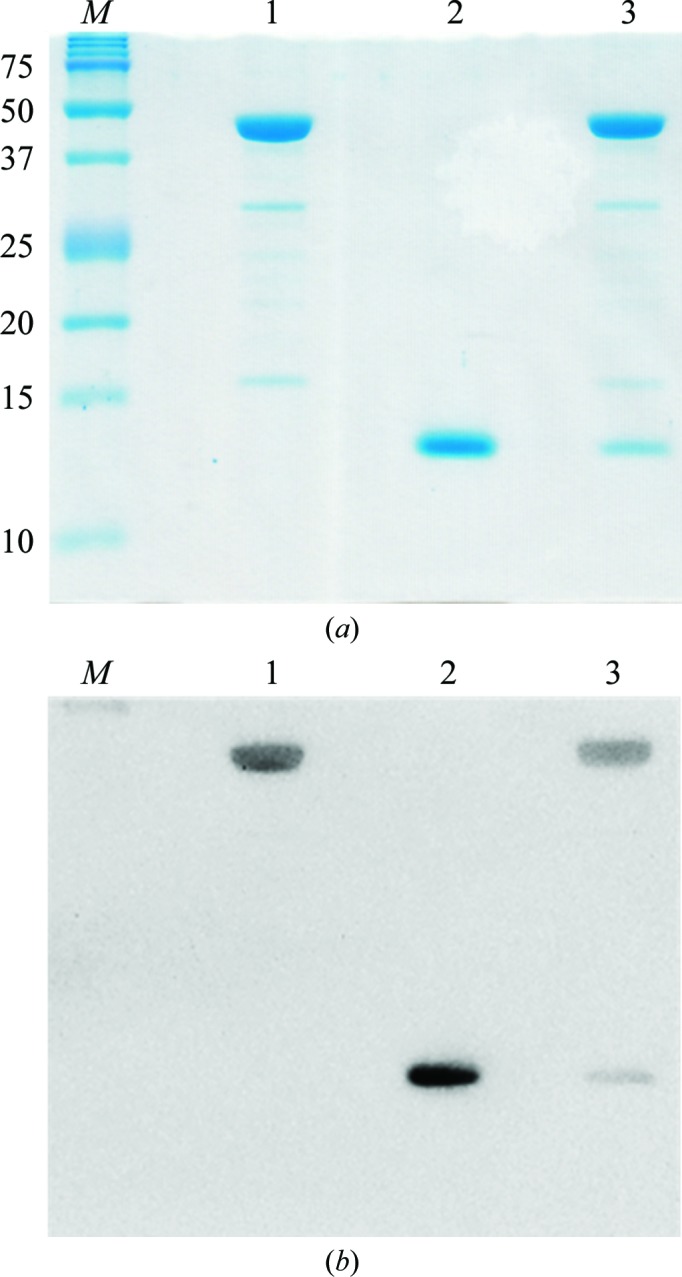
SDS–PAGE followed by Western blot analysis of Oxy and Fd complex crystals. (*a*) The dissolved crystals were verified by SDS–PAGE. Lane *M*, Precision Plus Protein Dual Color Standards (Bio-Rad; labelled in kDa); lane 1, Oxy solution used for crystallization; lane 2, Fd solution used for crystallization; lane 3, dissolved crystals of the complex. Approximately 3 µg protein was loaded per lane. (*b*) A Western blot stained with the anti-His antibody is shown; the lane numbers are the same as those in (*a*).

For crystallization experiments, Oxy_red_ and Fd_ox_ were mixed in a 1:3 molar ratio and then mixed with glycerol as an additive. Total protein concentration was adjusted to 15–30 mg ml^−1^ and the glycerol concentration was adjusted to 10%(*v*/*v*). Crystallization was performed using the hanging-drop vapour-diffusion method at 293 K. Drops consisting of 2 µl protein solution and 2 µl mother liquor were equilibrated against 400–600 µl reservoir solution. The initial crystallization conditions were screened using Crystal Screen, Crystal Screen 2, Crystal Screen Cryo and Index (Hampton Research, Laguna Hills, California, USA) and the Crystallization Kit for Protein Complexes (Sigma–Aldrich, St Louis, Missouri, USA). Several crystals were obtained using the Crystallization Kit for Protein Complexes condition No. 12 [0.1 *M* sodium cacodylate pH 6.5, 20% (*w*/*v*) PEG 3350]. To improve the crystallization conditions, the pH and precipitant concentration were assessed. Finally, plate-shaped crystals were obtained using 0.1 *M* sodium cacodylate pH 5.7, 14%(*w*/*v*) PEG 3350 (Table 1 ▶, Fig. 2 ▶). Crystal growth was observed after 1–2 d. SDS–PAGE and Western blot analysis were performed to verify the presence of both Oxy and Fd in these crystals. The crystals obtained in this study were dissolved in 5 m*M* Tris–HCl. The resulting protein, Oxy and Fd solutions were subjected to SDS–PAGE and stained with Coomassie Brilliant Blue. Although there were two bands corresponding to the sizes of Oxy and Fd, possible degradation peptides derived from Oxy were also detected (Fig. 3 ▶ *a*, lanes 1 and 3). After SDS–PAGE, the proteins were also transferred onto Sequi-Blot polyvinylidene difluoride (PVDF) membranes (Bio-Rad). Oxy and Fd were detected using anti-His antibody (GE Healthcare, Buckinghamshire, England) as the primary antibody and HRP-linked anti-mouse antibody (GE Healthcare) as the secondary antibody. Signals were visualized using the Luminescent Image Analyzer LAS-1000 Plus (Fujifilm, Tokyo, Japan). The hybridization signals for possible Oxy degradation peptides were not detected, while signals corresponding to the positions of Fd and Oxy were clearly detected in the obtained crystal (Fig. 3 ▶ *b*), indicating an Oxy–Fd binary complex.

### 2.3. Determination of the redox states of the crystals   

**Figure 4:**
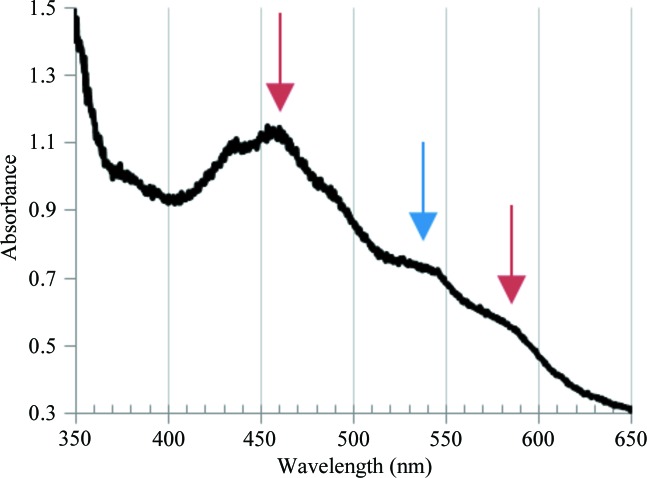
Absorption spectrum of binary-complex crystals. The blue arrow indicates the peak shoulder specific for reduced Oxy (530–540 nm) and the red arrows indicate those specific for oxidized Fd (460 and 570–590 nm).

To verify the redox state of each component in the crystals, the absorption spectrum of crystals of the binary complex was measured using a microspectrophotometer under a cryostream of nitrogen at 100 K (Chiu *et al.*, 2006 ▶). The microspectrophotometer system consisted of a deuterium tungsten halogen light (DT-MINI; Ocean Optics, Tokyo, Japan), Cassegrainian mirrors (Bunkoh-Keiki, Tokyo, Japan), an optical fibre and a linear CCD array spectrometer (SD2000; Ocean Optics). The crystals showed characteristic peaks at around 460 nm and peak shoulders at 525–540 and 570–590 nm (Fig. 4 ▶). In comparison with previous results (Nam *et al.*, 2002 ▶; Matsuzawa *et al.*, 2013 ▶), the peak at around 460 nm and the peak shoulder at 570–590 nm suggested that the Rieske [2Fe–2S] cluster in Fd was oxidized, because the oxidized form of Oxy shows a peak shoulder at a shorter wavelength such as 550–570 nm. On the other hand, the peak shoulder at 530–540 nm suggested that the Rieske [2Fe–2S] cluster of Oxy was reduced, because the reduced form of Fd shows a peak shoulder at a shorter wavelength such as 510–520 nm (Nam *et al.*, 2002 ▶; Matsuzawa *et al.*, 2013 ▶). Accordingly, the crystals were found to be composed of Oxy_red_ and Fd_ox_.

### 2.4. Data collection   

The crystals were cryocooled using liquid nitrogen in an anaerobic chamber with a mixture of 0.1 *M* sodium cacodylate pH 5.7, 14% PEG 3350 and 15% glycerol as a cryoprotectant.

X-ray diffraction data were collected on a MAR225 CCD detector at 100 K using synchrotron radiation of wavelength 1.0 Å on BL26B2 at SPring-8 (Harima, Japan) and were processed using *HKL*-2000 (Otwinowski & Minor, 1997 ▶). The crystals diffracted to 2.25 Å resolution and belonged to space group *P*2_1_, with unit-cell parameters *a* = 97.3, *b* = 81.6, *c* = 116.2 Å, α = γ = 90, β = 100.1°. The data-collection and processing statistics are shown in Table 2 ▶. The structure of the Oxy_ox_–Fd_ox_ binary complex (PDB entry 2de5; Ashikawa *et al.*, 2006 ▶) was used as a molecular-replacement model for *MOLREP* (Winn *et al.*, 2011 ▶; Vagin & Teplyakov, 2010 ▶). Although previous Oxy–Fd binary-complex crystals consisted of three molecules of the Fd monomer and one molecule of the Oxy trimer, the complex structure calculated from these crystals lacked one molecule of the Fd monomer and contained only two molecules of the Fd monomer per one Oxy trimer. After recalculation of this structure, the Matthews coefficient (*V*_M_; Matthews, 1968 ▶) was found to be 2.85 Å^3^ Da^−1^, indicating a solvent content of 56.8%.

A full description of the structure determination followed by interpretation of the structure–function relationship will be published elsewhere.

## Figures and Tables

**Table 1:** Crystallization conditions of Oxy, Fd and OxyFd NA, not applicable. CAR = carbazole.

			Treatment of the crystal formed			
	Condition	Space group	Reduction	CAR exposure	Air exposure	Reference
Oxy_ox_	Aerobic, hanging drop, 293K, 1719%(*v*/*v*) MPD, 0.1*M* MES pH 6.2	*P*2_1_3	NA	NA	NA	Nojiri *et al.* (2005 ▶)
Oxy_red_	Anaerobic, hanging drop, 293K, 30%(*v*/*v*) PEG MME 550, 0.05*M* MgCl_2_  6H_2_O, 0.1*M* HEPES pH 7.5	*P*6_5_	NA	NA	NA	Matsuzawa *et al.* (2013 ▶)
Fd_ox_	Aerobic, hanging drop, 278K, 1719%(*w*/*v*) PEG MME 2000, 0.2*M* (NH_4_)_2_SO_4_, 0.02*M* MgCl_2_  6H_2_O, 0.1*M* sodium acetate pH 4.6	*P*4_1_32	NA	NA	NA	Nam *et al.* (2005 ▶)
Oxy_ox_Fd_ox_	Aerobic, hanging drop, 293K, 12m*M* sodium dithionite, 14%(*w*/*v*) PEG MME 2000, 0.05*M* Bis-Tris pH 6.5	*P*2_1_	NA	NA	NA	Ashikawa *et al.* (2006 ▶)
Oxy_red_Fd_red_			Yes	NA	NA	
Oxy_ox_Fd_ox_CAR			NA	Yes	NA	
Oxy_red_Fd_red_CAR			Yes	Yes	NA	Ashikawa *et al.* (2012 ▶)
Oxy_ox_Fd_ox_O_2_			Yes	NA	Yes	
Oxy_ox_Fd_ox_O_2_CAR			Yes	Yes	Yes	
Oxy_red_Fd_ox_	Anaerobic, hanging drop, 293K, 14%(*w*/*v*) PEG 3350, 0.1*M* sodium cacodylate pH 5.7	*P*2_1_	NA	NA	NA	This study

**Table 2:** Crystal parameters and data-collection statistics Values in parentheses are for the highest resolution shell.

Space group	*P*2_1_
Unit-cell parameters (, )	*a* = 97.3, *b* = 81.6, *c* = 116.2, = = 90, = 100.1
Beamline	BL26B2, SPring-8
Wavelength ()	1.000
Rotation range per image ()	0.5
Total rotation range ()	360
Exposure time per image (s)	10
Crystal-to-detector distance (mm)	213.50
Resolution range ()	50.002.25 (2.332.25)
Total No. of reflections	579068
No. of unique reflections	82730 (7180)
Completeness (%)	97.0 (84.7)
Mosaicity ()	0.54 0.86
Average *I*/(*I*)	42.6 (3.5)
*R*_merge_ † (%)	6.8 (38.1)
Multiplicity	7.0 (5.4)
Overall *B* factor from Wilson plot (^2^)	46.9

† *R*_merge_ = 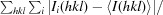 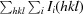, where *I_i_*(*hkl*) is the *i*th observation of reflection *hkl* and *I*(*hkl*) is the weighted average intensity for all observations of reflection *hkl*.

## References

[bb1] Ashikawa, Y., Fujimoto, Z., Noguchi, H., Habe, H., Omori, T., Yamane, H. & Nojiri, H. (2005). *Acta Cryst.* F**61**, 577–580. 10.1107/S1744309105014557 PMC1952320 16511100

[bb2] Ashikawa, Y., Fujimoto, Z., Noguchi, H., Habe, H., Omori, T., Yamane, H. & Nojiri, H. (2006). *Structure*, **14**, 1779–1789. 10.1016/j.str.2006.10.004 17161368

[bb3] Ashikawa, Y., Fujimoto, Z., Usami, Y., Inoue, K., Noguchi, H., Yamane, H. & Nojiri, H. (2012). *BMC Struct. Biol.* **12**, 15. 10.1186/1472-6807-12-15 PMC3423008 22727022

[bb4] Chiu, Y.-C., Okajima, T., Murakawa, T., Uchida, M., Taki, M., Hirota, S., Kim, M., Yamaguchi, H., Kawano, Y., Kamiya, N., Kuroda, S., Hayashi, H., Yamamoto, Y. & Tanizawa, K. (2006). *Biochemistry*, **45**, 4105–4120. 10.1021/bi052464l 16566584

[bb5] Gibson, D. T. & Parales, R. E. (2000). *Curr. Opin. Biotechnol.* **11**, 236–243. 10.1016/s0958-1669(00)00090-2 10851146

[bb6] Inoue, K. & Nojiri, H. (2014). *Biodegradative Bacteria*, edited by H. Nojiri, M. Tsuda, M. Fukuda & Y. Kamagata, pp. 181–205. Tokyo: Springer.

[bb7] Matsuzawa, J., Umeda, T., Aikawa, H., Suzuki, C., Fujimoto, Z., Okada, K., Yamane, H. & Nojiri, H. (2013). *Acta Cryst.* F**69**, 1284–1287. 10.1107/S1744309113026754 PMC3818054 24192370

[bb8] Matthews, B. W. (1968). *J. Mol. Biol.* **33**, 491–497. 10.1016/0022-2836(68)90205-2 5700707

[bb9] Nam, J.-W., Noguchi, H., Fujimoto, Z., Mizuno, H., Ashikawa, Y., Abo, M., Fushinobu, S., Kobashi, N., Wakagi, T., Iwata, K., Yoshida, T., Habe, H., Yamane, H., Omori, T. & Nojiri, H. (2005). *Proteins*, **58**, 779–789. 10.1002/prot.20374 15645447

[bb10] Nam, J.-W., Nojiri, H., Noguchi, H., Uchimura, H., Yoshida, T., Habe, H., Yamane, H. & Omori, T. (2002). *Appl. Environ. Microbiol.* **68**, 5882–5890. 10.1128/AEM.68.12.5882-5890.2002 PMC134387 12450807

[bb11] Nojiri, H. (2012). *Biosci. Biotechnol. Biochem.* **76**, 1–18. 10.1271/bbb.110620 22232235

[bb12] Nojiri, H., Ashikawa, Y., Noguchi, H., Nam, J.-W., Urata, M., Fujimoto, Z., Uchimura, H., Terada, T., Nakamura, S., Shimizu, K., Yoshida, T., Habe, H. & Omori, T. (2005). *J. Mol. Biol.* **351**, 355–370. 10.1016/j.jmb.2005.05.059 16005887

[bb13] Nojiri, H. & Omori, T. (2006). *Pseudomonas*, edited by J.-L. Ramos & R. C. Levesque, Vol. 5, pp. 107–145. New York: Springer.

[bb14] Otwinowski, Z. & Minor, W. (1997). *Methods Enzymol.* **276**, 307–326. 10.1016/S0076-6879(97)76066-X 27754618

[bb15] Parales, R. E. & Resnick, S. M. (2006). *Pseudomonas*, edited by J.-L. Ramos & R. C. Levesque, Vol. 4, pp. 287–340. New York: Springer.

[bb16] Senda, M., Kishigami, S., Kimura, S., Fukuda, M., Ishida, T. & Senda, T. (2007). *J. Mol. Biol.* **373**, 382–400. 10.1016/j.jmb.2007.08.002 17850818

[bb17] Vagin, A. & Teplyakov, A. (2010). *Acta Cryst.* D**66**, 22–25. 10.1107/S0907444909042589 20057045

[bb18] Winn, M. D. *et al.* (2011). *Acta Cryst.* D**67**, 235–242.

